# Diabetic kidney disease progression is associated with decreased lower-limb muscle mass and increased visceral fat area in T2DM patients

**DOI:** 10.3389/fendo.2022.1002118

**Published:** 2022-10-06

**Authors:** Xiaopu Lin, Zhenguo Chen, Haishan Huang, Jingyi Zhong, Lingling Xu

**Affiliations:** ^1^ Department of Huiqiao Medical Centre, Nanfang Hospital, Southern Medical University, Guangzhou, China; ^2^ Department of Endocrinology, Shenzhen Hospital, Southern Medical University, Shenzhen, China; ^3^ The Third School of Clinical Medicine, Southern Medical University, Guangzhou, China

**Keywords:** Sarcopenia, abdominal obesity, visceral fat area, diabetic kidney disease, type 2 diabetes mellitus

## Abstract

**Aim:**

This study aimed to explore the relationship between lower-limb muscle mass/visceral fat area and diabetic kidney disease (DKD) progression in patients with type 2 diabetes mellitus (T2DM).

**Methods:**

A total of 879 participants with T2DM were divided into 4 groups according to the prognosis of CKD classification from Kidney Disease: Improving Global Outcomes (KDIGO). Rectus femoris cross-sectional area (RFCSA) was measured through ultrasound, and visceral fat area (VFA) was evaluated with bioelectric impedance analysis (BIA).

**Results:**

T2DM patients with high to very high prognostic risk of DKD showed a reduced RFCSA (male *P* < 0.001; female *P* < 0.05), and an enlarged VFA (male *P* < 0.05; female *P* < 0.05). The prognostic risk of DKD was negatively correlated with RFCSA (*P* < 0.05), but positively correlated with VFA (*P* < 0.05). Receiver-operating characteristic analysis revealed that the cutoff points of T2DM duration combined with RFCSA and VFA were as follows: (male: 7 years, 6.60 cm2, and 111 cm2; AUC = 0.82; 95% CI: 0.78–0.88; sensitivity, 78.0%; specificity, 68.6%, P < 0.001) (female: 9 years, 5.05 cm2, and 91 cm2; AUC = 0.73; 95% CI: 0.66–0.81; sensitivity, 73.9%; specificity, 63.3%, P < 0.001).

**Conclusion:**

A significant association was demonstrated between reduced RFCSA/increased VFA and high- to very high-prognostic risk of DKD. T2DM duration, RFCSA, and VFA may be valuable markers of DKD progression in patients with T2DM.

**Clinical trial registration:**

http://www.chictr.org.cn, identifier ChiCTR2100042214

## Introduction

Diabetic kidney disease (DKD) is an important microvascular complication of diabetes, leading to increased mortality in diabetic patients (1). It has been reported that T2DM affects 8.2% of adults (2), 20%–40% of whom are expected to be diagnosed with DKD (3). The only treatment options for late stage DKD include dialysis or kidney transplantation, which are costly, significantly increasing personal and social burdens (4). Hence, identifying and managing the risk factors for DKD is of paramount importance in clinical practice.

Skeletal muscle constituting about 40% of body weight in healthy weight adults falls in quantity and quality with age (5). The mass of skeletal muscle also differs between the sexes. Sarcopenia characterized by gradual skeletal muscle strength and mass deterioration is also considered a complication of DM and has received increasing attention in recent years (6, 7). Many studies have shown that sarcopenia syndrome is commonly found in chronic kidney disease (CKD) patients, mainly those with end-stage kidney disease (ESKD) who received hemodialysis (8). Although previous studies have explored sarcopenia in DM or CKD (9, 10), whether it is associated with DKD is still unclear. No unified definition of sarcopenia has been recommended so far, and the consensus by the European Working Group on Sarcopenia in Older People (EWGSOP) is widely accepted (6, 11). The Asian Working Group for Sarcopenia (AWGS) further provided specific cutoff values for Asian population (12). The assessment of sarcopenia is complex and time-consuming, requiring simple techniques capable of monitoring changes in muscle mass as disease progresses. Douglas W. et al. demonstrated that ultrasound‐derived rectus femoris cross-sectional area (RFCSA) appeared to be a reliable index of total quadriceps volume, which was a measure of muscle mass (13). Mueller et al. showed that ultrasound might be a rapid and convenient method to assess sarcopenia (14).

Obesity has become a global health problem due to its associations with coronary artery disease, T2DM, nonalcoholic fatty liver disease, etc. (15, 16). Moreover, some studies have shown that abdominal obesity adversely affects renal prognosis, which is independent of diabetes (17, 18). Previous studies demonstrated that excessive visceral fat area (VFA) was related to insulin resistance and was a crucial risk factor for the development of T2DM compared with waist circumference or body mass index (BMI) (19, 20). The present study was designed to investigate the relationship between RFCSA/VFA and the prognostic risk of DKD, and to elucidate whether RFCSA/VFA was a marker for DKD progression.

## Materials and methods

### Study design

This controlled, open-label, cross-sectional trial was performed to explore the relationship between RFCSA/VFA and DKD progression. A total of 879 participants were enrolled at the Department of Endocrinology, Shenzhen Hospital, Southern Medical University, China, between March 2020 and December 2021.

Patients included were more than 18 years and were diagnosed with T2DM.

The exclusion criteria were listed as follows: acute complications of diabetes, such as hyperglycemic hyperosmolar coma, hypoglycemic coma, diabetic ketoacidosis and lactic acidosis; nondiabetic nephropathy; myasthenia or muscular atrophy caused by other factors, such as central and peripheral nervous system inflammatory or degenerative diseases, congenital/hereditary diseases, cerebrovascular diseases, craniocerebral trauma, and bone and joint diseases; and malignant tumors, chronic heart failure with decreased ejection fraction, severe liver disease, uncontrolled hypertension, and pregnancy.

The patients’ clinical data, such as sex, age, diabetes duration, BMI, blood pressure, history of alcohol consumption, smoking history, were recorded. Laboratory measurements, including blood urea nitrogen (BUN), creatinine (Cr), cystatin C (CysC), serum uric acid (SUA), blood lipid profile, glycosylated hemoglobin (HbA1c), fasting plasma glucose (FPG), fasting C-peptide (FCP), and fasting insulin (FINS), were tested after an 8-h fast. Also, 24-h urinary albumin excretion rate (UAER) and urinary albumin-to-creatinine ratio (UACR) were measured and recorded. Estimated glomerular filtration rate (eGFR) was calculated using CKD-EPI (21, 22). According to the prognosis of CKD classification from Kidney Disease: Improving Global Outcomes (KDIGO) 2020 Clinical Practice Guideline (23), the participants were divided into 4 groups as follows: low risk, moderate risk, high risk and very high-risk groups.

### RFCSA assessment using ultrasound

RFCSA was measured by ultrasonography using a 3-12 MHz transducer array (Philips Ultrasound, WA, USA) as previously described (24, 25). All measurements were made by the same sonographer. The patients were asked to keep relaxed, extend legs and to be in a supine position with upper body elevated by 30°. The point 60% of the distance from the anterior superior iliac spine to the superior border of the patella was located, and the ultrasound probe was placed perpendicularly along the superior part of the right thigh to obtain the transverse images of the RF (14).

### VFA assessment by BIA

Abdominal VFA was estimated using an Omron DUALSCAN BIA machine (Omron HDS-2000, Kyoto, Japan), which was a multifrequency impedance body composition analyzer. Eight-point tactile electrode method was utilized following the protocol. Resistance at five specific frequencies (1, 50, 250, 500 kHz, and 1 MHz) and reactance at three specific frequencies (5, 50 and 250 kHz) were measured to obtain the reading of VFA (cm^2^) on the screen. All measurements were performed by the same experienced researcher.

### Statistical analysis

Statistical analyses were performed using SPSS version 20.0 (SPSS Inc., Chicago, IL, USA). Descriptive data were expressed as mean ± standard deviation for continuous variables with a normal distribution and as median (interquartile range) for non-normal distribution variables. Categorical variables were summarized using percentage or frequency. Continuous data with normal distribution in different groups were compared using independent sample t test or one-way analysis of variance (ANOVA), whereas the Kruskal–Wallis test was performed for parameters with a skewed distribution. Pearson’s *χ* ^2^ test was employed to analyze categorical data. Spearman’s correlation analysis was used to explore the association between different prognostic risks of DKD and clinical characteristics (age, duration, TG, HbA1c, RFCSA, and VFA) of patients with T2DM stratified by sex. Multivariate logistic regression was performed to determine the risk factors for high-/very high-risk prognosis of DKD. Furthermore, receiver-operating characteristic (ROC) analysis was performed to determine the optimal cutoff points of diabetes duration, RFCSA and VFA for indicating high/very high prognostic risk of DKD in male and female patients respectively. All statistical analyses were 2-tailed and a *P* < 0 .05 was considered significant.

## Results

### Baseline characteristics of patients

In total, 941 T2DM patients underwent screening, and 879 participants were enrolled, as 62 were excluded based on exclusion criteria. Of these subjects, 270 patients (30.72%) were diagnosed with DKD according to KDIGO 2020 Clinical Practice Guideline (23). The patients were stratified into 4 groups according to KDIGO prognostic risk classification (low risk, moderate risk, high risk, and very high risk) (23). The baseline characteristics of the participants enrolled are presented in **Table 1**. Significant differences in sex (*P* < 0.05), age (*P* < 0.001), duration (*P* < 0.001), SBP (*P* < 0.001), DBP (*P* < 0.001), Cr (P < 0.001), BUN (*P* < 0.001), CysC (P < 0.001), SUA (*P* < 0.001), TG (*P* < 0.05), HDL (P < 0.05), HbA1c (P < 0.05), FPG (*P* < 0.001), FCP (*P* < 0.001), FINS (*P* < 0.05), UAER (*P* < 0.001), and UACR (*P* < 0.001) were observed among the groups. However, smoking, alcohol consumption, BMI, TC, and LDL displayed nonsignificant differences among the groups. Considering that the muscle content distribution was different between men and women, it was necessary to conduct statistical analysis for each sex. Male or female patients were then divided into two groups: high- to very high-risk and low- to moderate-risk groups. The results showed that RFCSA of the high- to very high-risk group was lower than that of the low- to moderate-risk group (male *P* < 0.001; female *P* < 0.05), whereas VFA of the high- to very high-risk group was higher than that of the low- to moderate-risk group (male *P* < 0.05; female *P* < 0.05) regardless of sex (**Table 2**).

**Table 1:** Clinical characteristics of T2DM patients with different prognosis risk of DKD.

	Low risk(n=609)	Moderately risk (n=174)(n=174)	High risk(n=50)	Very high risk(n=46)	*P*
**Sex(M/F)**	391/218	120/54	30/20	20/26	<0.05*
**Age (years)**	53.03 ± 12.02	53.29 ± 13.89	66.50 ± 9.44	61.72 ± 12.07	<0.001**
**Duration (years)**	5.0 (1.0, 10.0)	8.0 (1.0, 13.0)	10.0 (7.0, 17.0)	16.0 (8.0, 20.0)	<0.001**
**BMI (kg/m2)**	20.60 ± 4.77	21.55 ± 6.03	21.26 ± 6.53	19.52 ± 7.08	>0.05
**SBP (mmHg)**	126.60 ± 15.46	134.55 ± 19.32	136.42 ± 18.92	139.00 ± 19.65	<0.001**
**DBP (mmHg)**	78.04 ± 9.76	81.94 ± 12.99	78.18 ± 11.49	78.20 ± 11.36	<0.001**
**Alcohol (%)**	17.7%	18.4%	8.0%	10.9%	>0.05
**Smoking (%)**	27.8%	25.3%	16.0%	15.2%	>0.05
**BUN (mmol/L)**	4.78 ± 1.37	5.17 ± 1.69	7.35 ± 2.45	9.39 ± 3.66	<0.001**
**Cr (μmol/L)**	68.31 ± 16.18	77.25 ± 23.96	108.10 ± 26.40	153.33 ± 79.08	<0.001**
**CysC (mg/mL)**	0.91 ± 0.16	1.02 ± 0.24	1.74 ± 0.98	2.90 ± 2.44	<0.001**
**SUA (μmol/L)**	325.65 ± 90.39	374.12 ± 113.41	363.24 ± 103.55	362.11 ± 103.68	<0.001**
**TG (mmol/L)**	1.53(1.04, 2.30)	1.82(1.24,3.18)	1.67 (0.96, 2.87)	1.76(1.42, 2.88)	<0.001**
**TC (mmol/L)**	4.43 ± 1.44	4.61 ± 1.85	4.16 ± 1.20	4.47 ± 1.37	>0.05
**LDL (mmol/L)**	2.81 ± 1.08	3.59 ± 1.26	2.62 ± 1.08	2.61 ± 1.10	>0.05
**HDL (mmol/L)**	1.20 ± 0.34	1.08 ± 0.34	1.14 ± 0.36	1.16 ± 0.25	<0.05*
**HbA1C (%)**	9.28 ± 2.49	9.74 ± 2.38	9.01 ± 2.66	8.77 ± 2.14	<0.05*
**FPG (mmol/L)**	8.09 ± 2.94	9.09 ± 3.14	7.75 ± 3.30	7.61 ± 3.06	<0.001**
**FCP (ng/mL)**	2.03 ± 1.27	2.24 ± 1.20	2.78 ± 1.73	2.80 ± 1.57	<0.001**
**FINS (μU/mL)**	6.75(4.07, 11.53)	8.13(4.96, 13.05)	9.39(3.87, 16.94)	6.82(4.97, 11.61)	<0.05*
**UAER (mg/24h)**	7.80 (4.84, 14.62)	55.71 (36.48, 140.15)	66.08 (18.67, 196.64)	90.96 (43.97, 1502.05)	<0.001**
**UACR** **(mg/mmoL)**	0.68 (0.41, 1.40)	4.16 (2.09, 15.19)	6.09 (2.36, 32.31)	12.50 (4.15, 171.43)	<0.001**

Values were expressed as mean ± SD for normally distributed data and median with interquartile range for non-normally distributed data, or n (%). Differences among the groups were analyzed by ANOVA for normally distributed values and by the Kruskal-Wallis test for nonparametric values. Pearson’s χ2 test was employed to analyze categorical data. BMI, body mass index; SBP, systolic blood pressure; DBP, diastolic blood pressure; BUN, blood urea nitrogen; Cr, creatinine; CysC, Cystatin C; SUA, serum uric acid; TG, triglycerides; TC, total cholesterol; HDL, high-density lipoprotein; LDL, low-density lipoprotein; HbA1c, glycated hemoglobin; FPG, fasting plasma glucose; FCP, fasting C-peptide; FINS, fasting insulin; UAER, urinary albumin excretion rate; UACR, urinary albumin to creatinine ratio. *P < 0.05. **P < 0.001.

**Table 2:** RFCSA and VFA of T2DM patients with different prognosis risk of DKD.

	Low-Moderately risk	High-Very high risk	*P*
**N (Male)**	511	50	
**RFCSA (cm^2^)**	7.59 ± 2.61	6.21 ± 1.78	<0.001**
**VFA (cm^2^)**	103.1 ± 45.6	116.2 ± 33.4	<0.05*
**N (Female)**	272	46	
**RFCSA (cm^2^)**	5.58 ± 1.92	4.64 ± 1.44	<0.05*
**VFA (cm^2^) F**	86.4 ± 36.7	99.4 ± 40.0	<0.05*

Values were expressed as mean ± SD for normally distributed data. Differences between the groups were analyzed by student’s t-test for normally distributed values. RFCSA, rectus femoris cross-sectional area; VFA, visceral fat area. *P < 0.05. **P < 0.001.

### Correlation analysis between the prognostic risk of DKD and clinical parameters of patients with T2DM

Spearman’s correlation was conducted to analyze the relationship between the prognostic risk of DKD and clinical parameters of male and female patients separately, and similar findings were noted. The results showed that the prognostic risk of DKD was negatively correlated with RFCSA (male *r* = − 0.138, *P* < 0.05; female *r* = − 0.194, *P* < 0.05), and positively correlated with age (male *r* = 0.210, *P* < 0.001; female *r* = 0.223, *P* < 0.001), duration (male *r* = 0.291, *P* < 0.001; female *r* = 0.212, *P* < 0.001), TG (male *r* = 0.103, *P* < 0.05; female *r* = 0.124, *P* < 0.05), and VFA (male *r* = 0.139, *P* < 0.05; female *r* = 0.144, *P* < 0.05). However, no significant association was observed between HbA1c and the prognostic risk of DKD in male or female patients with T2DM (**Table 3**).

**Table 3:** Spearman’s correlation analysis of different prognosis risk of DKD with Clinical characteristics in T2DM patients stratified by gender.

		r	*P*
**Male**	**Age**	0.210	<0.001**
	**Duration**	0.291	<0.001**
	**TG**	0.103	<0.05*
	**HbA1C**	-0.060	>0.05
	**RFCSA**	-0.138	<0.05*
	**VFA**	0.139	<0.05*
**Female**	**Age**	0.223	<0.001**
	**Duration**	0.212	<0.001**
	**TG**	0.124	<0.05*
	**HbA1C**	0.039	>0.05
	**RFCSA**	-0.194	<0.05*
	**VFA**	0.144	<0.05*

TG, triglycerides; HbA1c, glycated hemoglobin; RFCSA, rectus femoris cross-sectional area; VFA, visceral fat area. *P < 0.05. **P < 0.001.

### Multivariate logistic regression between the prognostic risk of DKD and clinical variables of patients with T2DM

Age and TG were excluded from multivariate logistic regression due to high inter-correlation between age and duration (*P* < 0.001, data not shown) and between VFA and TG (*P* < 0.001, data not shown). We performed multivariate logistic regression analysis using the prognostic risk of DKD as dependent variable (high-risk and very high-risk group defined as “1”, and low-risk and moderate-risk group defined as “0”), and duration, RFCSA and VFA as independent variables. As shown in **Table 4**, duration (β 1.11, 95% CI 1.07–1.16, *P* < 0.001), RFCSA (β 0.69, 95% CI 0.57–0.83, *P* < 0.001), and VFA (β 1.01, 95% CI 1.00–1.02, *P* < 0.05) was found to be significantly associated with high- to very high-risk prognosis of DKD in male patients with T2DM. Similarly, duration (β 1.04, 95% CI 1.01–1.07, *P* < 0.001), RFCSA (β 0.73, 95% CI 0.59–0.91, *P* < 0.05), and VFA (β 1.01, 95% CI 1.00–1.02, *P* < 0.05) was shown to be significantly linked with high- to very high-risk prognosis of DKD in female T2DM patients (**Table 4**).

**Table 4:** Risk factors for high-/very high-risk prognosis of DKD in multivariate logistic regression.

	Independent variables	β (95% Cl)	*P*
**Male**	**Duration**	1.11(1.07, 1.16)	<0.001**
	**RFCSA**	0.69 (0.57, 0.83)	<0.001**
	**VFA**	1.01 (1.00, 1.02)	<0.05*
**Female**	**Duration**	1.04(1.01, 1.07)	<0.05*
	**RFCSA**	0.73 (0.59, 0.91)	<0.05*
	**VFA**	1.01 (1.00, 1.02)	<0.05*

RFCSA, rectus femoris cross-sectional area; VFA, visceral fat area. *P < 0.05. **P < 0.001.

### ROC analysis

**Figure 1:**
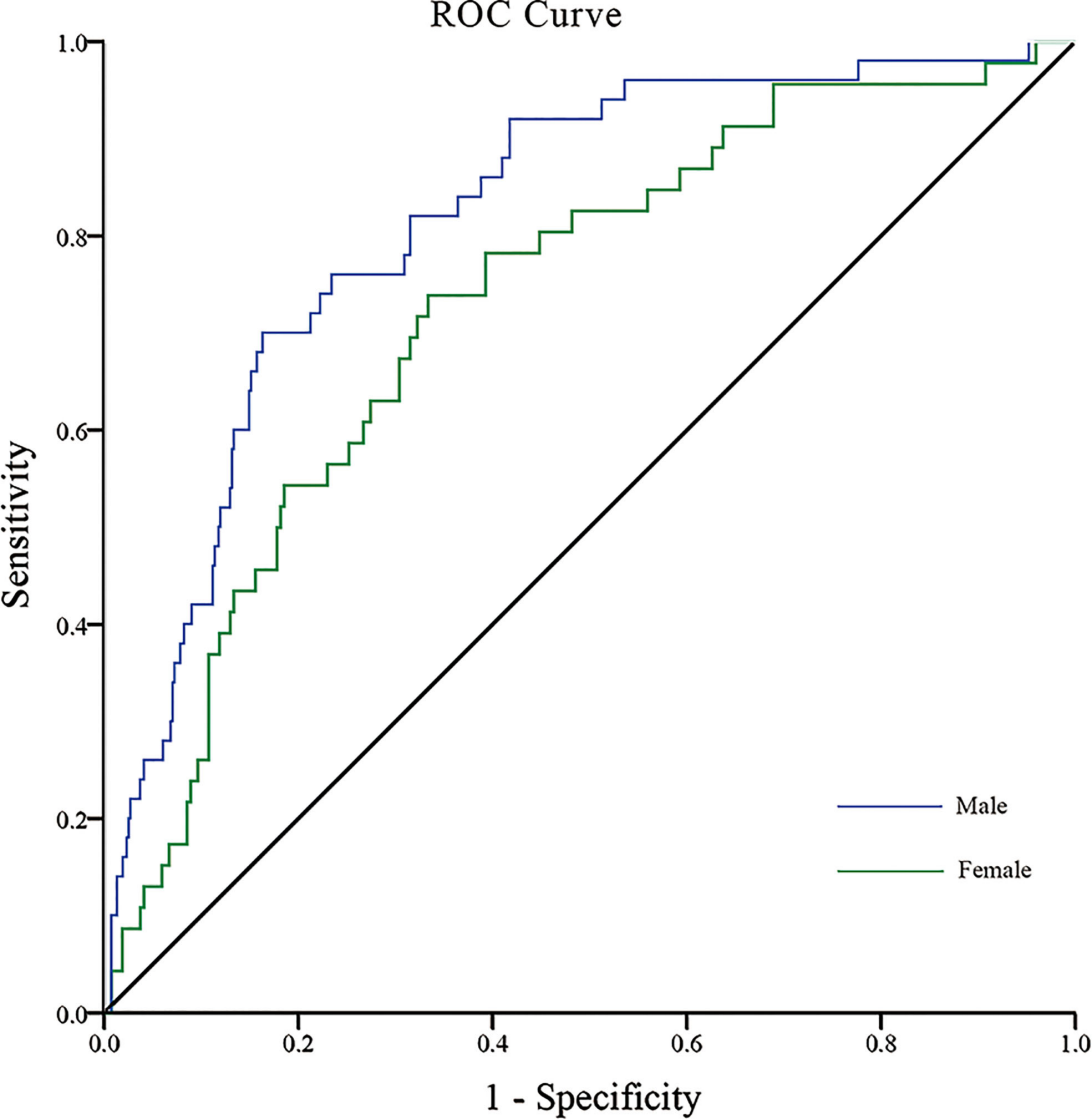
ROC analysis of T2DM duration combined with RFCSA and VFA to predict high-/very high-risk prognosis of DKD in male/female T2DM patients. [Male (blue): AUC=0.82; 95% CI: 0.78–0.88; Sensitivity 78.0%, Specificity 68.6%; *P* < 0.001] [Female (green): AUC=0.73; 95% CI: 0.66–0.81; Sensitivity 73.9%, Specificity 63.3%, P < 0.001].

We performed ROC analysis to investigate the optimal cutoff points for diabetes duration, RFCSA and VFA, which could be used to distinguish a high- to very high-risk prognosis of DKD. Multivariate logistic regression analysis was carried out to assess the predictive capability of the combined parameters of diabetes duration, RFCSA and VFA, which were used as independent variables for multivariable ROC analysis. For T2DM male patients, the cutoff values of diabetes duration, RFCSA and VFA were revealed as 7 years, 6.60 cm^2^ and 111 cm^2^, respectively, with an AUC of 0.82 (95% CI: 0.78–0.88), a sensitivity of 78.0%, and a specificity of 68.6% (*P* < 0.001) (**Figure 1** blue). For T2DM female patients, the cutoff of diabetes duration, RFCSA and VFA were 9 years, 5.05 cm^2^ and 91 cm^2^, respectively, the AUC was 0.73 (95% CI: 0.66–0.81), the sensitivity was 73.9%, and the specificity was 63.3% (*P* < 0.001) (**Figure 1** green).

## Discussion

DKD is a major cause of CKD worldwide and brings enormous economic burden to patients and society (26). In addition to the use of medication to control hyperglycemia and hypertension, modifying other related factors is of great importance for the management of DKD patients. Sarcopenia is a frequent condition reported in CKD patients and is considered to be linked with an increased risk of hospitalization and all-cause mortality (27). Previous studies have shown that sarcopenia reflects progressive and cumulative effects of CKD on skeletal muscle (13, 28). Abdominal obesity is a risk factor for multiple complications of diabetes. Heng Wan et al. showed that abdominal obesity was strongly associated with DKD (29).

In the present study, the patients were divided into two groups (high- to very high-risk group and low- to moderate-risk group) to explore the relationship between RFCSA/VFA and the prognostic risk of DKD. The results showed an obviously reduced RFCSA in the high- to very high-risk group compared with the low- to moderate-risk group. Although sarcopenia has been extensively explored in patients with diabetes or CKD (9, 30, 31), the changes of RFCSA in DKD patients has not yet been reported. Many studies have shown that the incidence rate of sarcopenia in ESKD patients is higher than that in patients with early-stage renal disease, which is consistent with our results (8). Some studies reported that abdominal obesity, compared with general obesity, had a greater impact on the risk of DKD (32, 33). Chin-Hsiao Tseng demonstrated a close and independent association between abdominal obesity and elevated UAER in female patients with diabetes but not in male diabetic patients (34). Our results showed an enlarged VFA in high- to very high-risk male and female DKD patients.

Furthermore, our study showed that the prognostic risk of DKD was positively correlated with age, duration and TG, which were recognized as important factors influencing the progression of DKD. A systematic review and meta-analysis of 20 cohorts comprising 41,271 individuals showed that the independent risk factors for DKD development were duration, age, smoking status, HbA1c, TG, HDL-C, BMI, SBP, UACR, and eGFR (35). In the present study, no relationship was established between HbA1c and DKD, which was different from the conclusions of previous studies (36). This discrepancy could be explained by the fact that HbA1c only reflected glycemic control in the recent 3 months. In addition, another explanation might be that some DKD patients were complicated with renal anemia, resulting in lower HbA1c concentration compared with the actual level.

This study also showed that the prognostic risk of DKD was negatively correlated with RFCSA. The exact underlying mechanism has not been fully elucidated. However, abnormal renal function and hyperglycemia are considered essential factors for sarcopenia in patients with DKD. Firstly, sarcopenic obesity, a combination of sarcopenia and obesity, reflects a vicious link between insulin resistance and sarcopenia. Obesity-induced insulin resistance triggers a series of events that lead to a decrease in muscle glucose supply and quantitative and qualitative deterioration of muscles, further enhancing insulin resistance and creating a vicious cycle (37). Secondly, accumulation of advanced glycation end-products (AGEs) and diabetic vasculopathy may also impair muscle mass and strength, leading to sarcopenia (38–40). Thirdly, with the worsening of renal function, sarcopenia occurs due to accelerated protein catabolism, and reduced energy and protein intake during dialysis (8).

The relationship between DKD and abdominal obesity was investigated in many previous studies. A meta-analysis, including 2205 patients with VFA measurements from 3 cross-sectional studies, demonstrated that VFA was associated with greater odds of DKD in patients with type 2 diabetes (34). Asakawa H et al. showed that VFA level was significantly higher in patients with DKD than those without DKD (41). However, some studies showed the contradictory conclusions. Man et al. (42) found that abdominal obesity had no association with DKD in patients with T2DM. Therefore, the relationship between abdominal obesity and DKD deserves further investigation. The present study found that VFA was positively correlated with the prognostic risk of DKD. Although the mechanisms underlying the linking between DKD and abdominal obesity are still unclear, several hypotheses may be proposed. Firstly, excessive visceral fat accumulation leads to systemic inflammation, which may contribute to a cascade of events such as insulin resistance, oxidative stress, and renal damage (43, 44). Secondly, the renin–angiotensin system (RAS) is activated by adipose tissue, which changes sodium retention and renal hemodynamics, ultimately leading to renal damage (45, 46). Thirdly, other metabolic syndromes that are associated with obesity also play an important role in the occurrence and development of DKD (47, 48).

Based on the results of this study, we suggested that the loss of lower-limb muscle mass and the increase in VFA were closely related to the progression of DKD. The prognostic risk of DKD was high or very high for male T2DM patients, with a duration of more than 7 years, a RFCSA of less than 6.60 cm^2^, and a VFA of more than 111 cm^2^. The prognostic risk of DKD was also high or very high for female T2DM patients, with a duration being more than 9 years, a RFCSA being less than 5.05 cm^2^, and a VFA being more than 91 cm^2^. Therefore, we speculated that the modified lifestyle to increase skeletal muscle mass and reduce visceral fat accumulation might delay the progression of DKD in patients with T2DM.

This study had some limitations. Firstly, certain confounding factors, such as the level of physical activity and the use of anti-diabetes medication, might also influence the results of the study. Secondly, the current conclusion was summarized from a cross-sectional trial. Thirdly, VFA was measured using a novel BIA device that has yet only received limited validation (49) rather than a more accurate and reliable method such as computed tomography (CT).

## Conclusions

The lower-limb muscle mass of T2DM patients decreased whereas VFA increased with the progression of DKD. The prognostic risk of DKD was negatively correlated with RFCSA but positively correlated with VFA. T2DM duration, RFCSA and VFA were found to be markers of DKD progression. Based on the conclusion of this study, for patients who have not developed DKD or are in the early stage of DKD, individualized lifestyle guidance (including diet and exercise) and reasonable hypoglycemic medicine selection should be given to increase muscle content and reduce abdominal fat, which may delay the occurrence and progress of DKD. In the future, cohort study and fundamental research are needed to verify the viewpoint and further explore relevant mechanisms.

## Data availability statement

The original contributions presented in the study are included in the article/supplementary material. Further inquiries can be directed to the corresponding author.

## Ethics statement

The studies involving human participants were reviewed and approved by Medical ethics committee of Shenzhen Hospital, Southern Medical University (Approval No. NYSZYYEC20200035). The patients/participants provided their written informed consent to participate in this study.

## Author contributions

Each author has made an important scientific contribution to the study and is thoroughly familiar with the primary data. XL and ZC carried out the clinical studies, participated in the statistical analysis and drafted the manuscript. HH and JZ carried out the data acquisition, participated in the manuscript preparation and literature research. LX conceived of the study, and participated in its design and helped to review the manuscript. All authors listed have read the complete manuscript and have approved submission of the paper.

## Funding

This study was supported by grant from the National Natural Science Foundation of China (No. 82270895), Science and Technology Planning Project of Shenzhen (No. JCYJ20210324130204011) and Young Scientific Talents Research Project of China Endocrine and Metabolism (No.2021-N-03).

## Conflict of interest

The authors declare that the research was conducted in the absence of any commercial or financial relationships that could be construed as a potential conflict of interest.

## Publisher’s note

All claims expressed in this article are solely those of the authors and do not necessarily represent those of their affiliated organizations, or those of the publisher, the editors and the reviewers. Any product that may be evaluated in this article, or claim that may be made by its manufacturer, is not guaranteed or endorsed by the publisher.
